# Biomarkers of alloimmune events in pediatric kidney transplantation

**DOI:** 10.3389/fped.2022.1087841

**Published:** 2023-01-20

**Authors:** Kyle A. Deville, Michael E. Seifert

**Affiliations:** Division of Pediatric Nephrology, Department of Pediatrics, University of Alabama Heersink School of Medicine, Birmingham, AL, United States

**Keywords:** biomarkers, alloimmunity, acute rejection, pediatrics, kidney transplantation

## Abstract

Alloimmune events such as the development of *de novo* donor-specific antibody (dnDSA), T cell-mediated rejection (TCMR), and antibody-mediated rejection (ABMR) are the primary contributors to kidney transplant failure in children. For decades, a creatinine-based estimated glomerular filtration rate (eGFR) has been the non-invasive gold standard biomarker for detecting clinically significant alloimmune events, but it suffers from low sensitivity and specificity, especially in smaller children and older allografts. Many clinically “stable” children (based on creatinine) will have alloimmune events known as “subclinical acute rejection” (based on biopsy) that merely reflect the inadequacy of creatinine-based estimates for alloimmune injury rather than a distinct phenotype from clinical rejection with allograft dysfunction. The poor biomarker performance of creatinine leads to many unnecessary surveillance and for-cause biopsies that could be avoided by integrating non-invasive biomarkers with superior sensitivity and specificity into current clinical paradigms. In this review article, we will present and appraise the current state-of-the-art in monitoring for alloimmune events in pediatric kidney transplantation. We will first discuss the current clinical standards for assessing the presence of alloimmune injury and predicting long-term outcomes. We will review principles of biomarker medicine and the application of comprehensive metrics to assess the performance of a given biomarker against the current gold standard. We will then highlight novel blood- and urine-based biomarkers (with special emphasis on pediatric biomarker studies) that have shown superior diagnostic and prognostic performance to the current clinical standards including creatinine-based eGFR. Finally, we will review some of the barriers to translating this research and implementing emerging biomarkers into common clinical practice, and present a transformative approach to using multiple biomarker platforms at different times to optimize the detection and management of critical alloimmune events in pediatric kidney transplant recipients.

## 1. Introduction

Kidney transplantation is the optimal treatment for children with kidney failure, providing improved quality of life along with reduced morbidity and mortality compared to long-term dialysis (1). Despite advances in surgical techniques, infection surveillance, and immunosuppression modalities in the modern era, mean kidney allograft survival has remained static at no more than 10–15 years for most pediatric recipients (2). Subsequent transplantation is often desired, albeit with longer wait times and increased immunologic risk due to the potential for human leukocyte antigen (HLA) sensitization from the prior transplant. As in adult kidney transplant recipients, alloimmune events such as T cell-mediated rejection (TCMR), the development of *de novo* anti-HLA donor-specific antibodies (dnDSA), and subsequent active antibody-mediated rejection (ABMR) remain the biggest contributors to kidney allograft failure in children (3). The incidence of acute rejection is 10%–20%, and while early treatment with escalated immunosuppression improves allograft survival for some, others are left with accelerated chronic allograft injury in the form of interstitial fibrosis, tubular atrophy, transplant glomerulopathy, and vasculopathy with detrimental impacts on allograft survival (4–7).

Modern day immunosuppressive regimens and HLA matching attempt to decrease risk for alloimmune events, but are still blunt instruments that do not account for interpatient variability in immune responsiveness and absorption of immunosuppressive medications, to say nothing of unpredictable medication adherence that afflicts many recipients in the high-risk adolescent age window (8). Adding to this challenge, the current monitoring standards for alloimmune events are either invasive and lack reproducibility (e.g., surveillance and for-cause kidney transplant biopsies), or are non-invasive biomarkers that have inadequate sensitivity/specificity and are elevated late in the alloimmune injury process once significant damage has already been established (e.g., creatinine-based estimates of kidney transplant function or proteinuria) (9–11).

The presence of alloimmune events prior to overt changes in creatinine-based estimated glomerular filtration rate (eGFR) or proteinuria has been coined “subclinical rejection,” which is somewhat of a misnomer since this entity has been associated with increased rates of subsequent dnDSA, clinical acute rejection (e.g., rejection with detectable allograft dysfunction), and allograft failure (12–15). In contrast, serum creatinine can be altered by a multitude of other non-alloimmune insults including hypovolemia, infection, calcineurin inhibitor toxicity, and urinary tract obstruction, leading to unnecessary for-cause biopsies in patients with apparent allograft dysfunction (16). Therefore, creatinine-based eGFR has relatively poor biomarker performance, with only 50%–60% sensitivity and specificity for alloimmune injury (17). Thus, there exists an unmet clinical need for non-invasive biomarkers with superior performance to creatinine-based eGFR that can allow early detection of alloimmune events and improve long-term outcomes in pediatric kidney transplant recipients. This review article will highlight key aspects of biomarker medicine and its application to kidney transplant diagnostics, then discuss select candidate biomarkers that are poised to address this difficult challenge in pediatric kidney transplantation.

## 2. Principles of biomarker medicine: Identity, biology, application, and performance

In 2015, the Joint Leadership Council of the Food and Drug Administration (FDA) and the National Institutes of Health (NIH) developed the Biomarkers, EndpointS, and other Tools (BEST) resource to harmonize the terms used in translational studies related to clinical endpoints and biomarkers (18). They developed a consensus definition of a biomarker as a “defined characteristic that is measured as an indicator of normal biologic processes, pathologic processes, or responses to an exposure or intervention.” They emphasized the importance of a *standardized biomarker description*: a succinct but comprehensive summary intended to correctly identify the biomarker, describe its biologic plausibility (i.e., relevance to the disease or condition), and define its measurement method. The identity of the biomarker simply includes its specific name or unique identifier, source material (e.g., urine), and type (e.g., molecular/protein). Biologic plausibility indicates the intended context of use by detailing how the biomarker reflects a biological pathway associated with the disease of interest (e.g., biomarker X increases at the time of biopsy-proven T cell-mediated rejection). Finally, the measurement method describes how the biomarker is to be measured along with its units of quantification (e.g., measured by ELISA in pg/mL). An example biomarker description would be: urine CXCL9 protein (measured in pg/mL by ELISA) has been shown to increase at the time of T cell-mediated rejection (19).

The BEST working group further subdivided biomarkers into seven broad categories based on clinical utility: diagnostic, monitoring, pharmacodynamics/response, predictive, prognostic, susceptibility/risk, and safety (18). *Diagnostic biomarkers* are used to confirm presence of a disease or identify one of its subtypes. They are important for identifying patients in need of treatment or confirming eligibility for participation in clinical trials. Among the biomarker subcategories, diagnostic biomarkers are most commonly evaluated against a gold standard diagnostic test (e.g., traditional histology in kidney transplantation) using performance metrics such as sensitivity and specificity. Performance assessment of diagnostic biomarkers will be reviewed in more detail in subsequent paragraphs. *Monitoring biomarkers* are used to assess current status of a disease and are often repeated serially over time. They can indicate progression or improvement of disease, especially in response to an intervention. A *response biomarker* indicates that a biological response has occurred after an intervention and has some overlap with monitoring biomarkers. An important type of response biomarker is a surrogate endpoint, which is often used to increase efficiency of clinical trials and drug development by serving as a reliable indicator for a subsequent hard clinical endpoint (20).

*Predictive biomarkers* identify patients with a disease of interest that are more likely to respond to an intervention, and can be used in precision medicine approaches to assign patients to different treatment groups based on their predicted efficacy (21). In contrast, *prognostic biomarkers* identify patients with a disease of interest that are more likely to experience a certain outcome regardless of the presence or absence of an intervention. As such, they are typically measured at baseline or “time-zero” when they indicate the likelihood of a future clinical event of interest (22). *Susceptibility/risk biomarkers*, by comparison, identify patients without clinically apparent disease that are more likely to develop the disease of interest at some point in the future. They are often used to enrich clinical trials with clinically stable patients that have increased risk to develop the disease under study (23). Finally, a *safety biomarker* is measured in the context of an intervention to indicate the likelihood of toxicity or an adverse effect. They can be used in isolation to predict the likelihood of an adverse effect, or serially to alter ongoing therapies to limit toxicity (24). A summary of all seven biomarker classes with examples in kidney transplantation is presented in Table 1.

**Table 1:** Classes of biomarkers in kidney transplantation. The seven classes of biomarkers in the FDA-NIH BEST resource are listed with clinical and investigational examples of each type and corresponding references. Some examples can be used as multiple types of biomarkers based on available data.

Biomarker Type	Examples In Transplantation	Reference Number
Diagnostic	Urine CXCL9/10, plasma/urine dd-cfDNA, gene expression profiling, urine mRNA	(17*, 19, 25–29*^30^–^38^*, 39)
Monitoring	Urine CXCL9/10, GFR, proteinuria, viral PCR	(17, 35–38*)
Predictive	Traditional and molecular histology, CD28+ T memory cells	(6, 21, 40–42)
Prognostic	Urine CXCL9/10, urine CCL2, dd-cfDNA, *dn*DSA, gene expression profiling, GFR, proteinuria	(17*, 43*, 25–38*)
Response	GFR, proteinuria, urine CXCL9/10, gene expression profiling	(17*, 19, 26)
Safety	Tacrolimus trough levels, viral PCR	(24*, 44)
Susceptibility	Urine CXCL9/10, *dn*DSA	(35–38*)

* Represent biomarker studies that included children.

In the context of pediatric kidney transplantation, one can place these different biomarker categories on a post-transplant timeline that reflects their optimal clinical utility. In the early period post-transplant, risk/susceptibility biomarkers give insight into which clinically stable patients are at increased risk for developing alloimmune responses that lead to acute rejection. For example, patients with normal/stable eGFR may develop early *de novo* DSA years before an episode of ABMR occurs (43). Diagnostic biomarkers are used serially throughout the post-transplant timeline to detect alloimmune events, and include analytes such as plasma donor-derived cell-free DNA and urine CXCL9/10 in patients with subclinical TCMR (25, 45–47). Response and monitoring biomarkers include traditional clinical assessments, such as eGFR and proteinuria, that are trended over time to indicate a successful response to treatment of TCMR or progression of chronic allograft injury, respectively (48, 49). However, a recent meta-analysis of studies with follow-up biopsies highlighted the poor performance of creatinine as a response biomarker for adequate treatment of TCMR (50, 51). Once an episode of late clinical acute rejection has occurred (e.g., TCMR at 3 years post-transplant), predictive biomarkers such as the Banff endarteritis (v) score may identify a patient that would benefit from anti-thymocyte globulin in addition to pulse intravenous methylprednisolone (52). In this same patient with high-grade TCMR, prognostic biomarkers such as biopsy-derived gene expression profiling can be used to determine risk for future allograft failure (52). Finally, tacrolimus trough levels, absolute neutrophil counts, and BK viral loads are commonly used safety biomarkers that are indicators of toxicity or adverse effects of immunosuppressive therapies (53). A summary of all seven biomarker classes with examples in kidney transplantation is presented in Table 1.

Assessing the performance of a diagnostic biomarker against an accepted gold standard is critical to understand its context of use (11, 54). The most frequently used biomarker performance metrics in kidney transplantation are sensitivity, specificity, positive and negative predictive values (PPV and NPV), accuracy, and area under the receiver operating characteristic curve (AUROC) - all referenced against a traditional histology diagnosis (18). Notably, while traditional histology is the reference gold standard for diagnosing acute rejection, it also suffers from modest sensitivity and lack of reproducibility between different interpreting pathologists (55). Recent studies have argued that molecular pathology, which uses biopsy-derived gene expression patterns as diagnostic tools, may be more sensitive and reproducible reference standard for detecting alloimmune events compared to traditional histology (40). Further discussion on this important topic is beyond the scope of this review.

Sensitivity refers to the ability of a diagnostic biomarker to correctly identify patients with actual disease. Sensitivity is the true positive (TP) rate: the probability that patients with disease will test positive for a given biomarker. In mathematical form, sensitivity = TP/[TP + false negatives (FN)]. A diagnostic biomarker with 100% sensitivity tests positive in all patients with the disease (as well as some patients without disease), but only tests negative in patients without disease. A biomarker with 75% sensitivity will still misclassify 25% of people with actual disease as normal/stable- a 25% false negative (FN) rate. While high sensitivity shows that a biomarker can diagnose the majority of patients with disease, it does not account for its performance in patients without disease, including the rate of false positive (FP) tests. PPV broadens our understanding of a biomarker's sensitivity and its “real-world” performance in a population with and without disease; it refers to the probability that patients with a positive biomarker test actually have disease. In mathematical form, PPV = TP/(TP + FP). Unlike sensitivity, it depends heavily on the prevalence of the disease of interest in the test population - achieving high PPV is easier in populations (or case-control studies) where the disease is more common and the FP rate will naturally be much lower than the TP rate (11). A diagnostic biomarker with 100% PPV means there will be no FP tests - all patients that test positive will have the disease. Therefore, a biomarker with high sensitivity but relatively low PPV will correctly diagnose most patients with disease, but at the expense of a high FP rate that subjects additional patients without disease to unnecessary interventions and stress. In kidney transplantation, since the prevalence of acute rejection is relatively low most diagnostic biomarkers that achieve high sensitivity will also have low PPV and produce a number of FP tests as a result. The number of excess biopsies performed may be an acceptable tradeoff, if the clinician believes that identifying and treating every episode of acute rejection will improve long-term transplant outcomes (51).

In contrast, specificity refers to the ability of a diagnostic biomarker to correctly identify stable patients without actual disease. Specificity is the true negative (TN) rate: the probability that patients without disease will test negative for a given biomarker. In mathematical form, specificity = TN/(TN + FP). A diagnostic biomarker with 100% specificity tests negative in all patients without the disease (as well as some patients with disease), but only tests positive in patients with disease. A biomarker with 80% specificity will still misclassify 20% of stable patients as having disease - a 20% FP rate. However, as with sensitivity, a diagnostic biomarker with high specificity does not account for the FN rate in patients with disease that are left undetected and untreated. NPV provides more real-world context to the specificity of a diagnostic biomarker in patients with and without disease. NPV refers to the proportion of all patients with a negative biomarker test that actually do not have the disease. In mathematical form, NPV = TN/(TN + FN). As with PPV, the prevalence of the disease in the test population is important - achieving high NPV is easier when the disease is less common (e.g., most cohort studies) and the false negative rate is likely to be much lower than the true negative rate (11). A diagnostic biomarker with 100% NPV means there will be no FN tests - all patients that test negative will be actually free of the disease. Therefore, a biomarker with high specificity but relatively low NPV will identify all stable patients without disease, but at the expense of misclassifying some patients with actual disease as stable (FN) that in fact should be treated. This may be acceptable if the context of use for the diagnostic biomarker is to perform fewer invasive biopsies and avoid unnecessary treatments in stable patients, and the consequences of untreated disease are relatively low (26). Since the prevalence of acute rejection is relatively low in pediatric kidney transplantation, most diagnostic biomarkers that achieve high specificity will also have high NPV and produce a low number of FN tests as a result (56). Put another way, the burden of achieving high specificity and NPV is easier than achieving high sensitivity and PPV for diagnostic biomarkers of acute rejection.

Diagnostic accuracy or area under the receiver operating characteristic curve (AUROC) are commonly used metrics to assess the overall performance of a diagnostic biomarker. Accuracy is the probability that a diagnostic biomarker will correctly identify both stable and diseased patients. Mathematically, accuracy = (TP + TN) total number of cases. A diagnostic accuracy of 80% means that most patients will be correctly classified as stable or diseased by their biomarker result. AUROC is generated by plotting the sensitivity against 1- specificity for each potential cutoff value for a given biomarker. When the AUROC approaches 0.5 or less, the diagnostic biomarker performs no better than a chance flip of a coin. The closer the AUROC is to 1.0, the better the biomarker performs across a range of cutoff values. While accuracy and AUROC are good overall measures of diagnostic performance, they can overestimate the performance of a biomarker when the size of stable and diseased groups is unbalanced, as is almost certainly the case in any cohort study of acute rejection in kidney transplantation. Specifically, combining high accuracy in a large group of stable patients with low accuracy in a comparatively small group of diseased patients can mask the biomarker's poor performance in diseased patients. Balanced accuracy may be a more appropriate assessment of the overall performance of a diagnostic biomarker for acute rejection and other alloimmune events that have a relatively low prevalence (41). Balanced accuracy is defined simply as the average of the sensitivity and specificity, and incorporates the average accuracy from both stable and diseased subgroups. We recommend reporting a complete set of metrics (sensitivity, specificity, PPV, NPV, balanced accuracy, and AUROC) in any biomarker study to provide a comprehensive assessment of diagnostic performance that is comparable to other studies.

**Figure 1:**
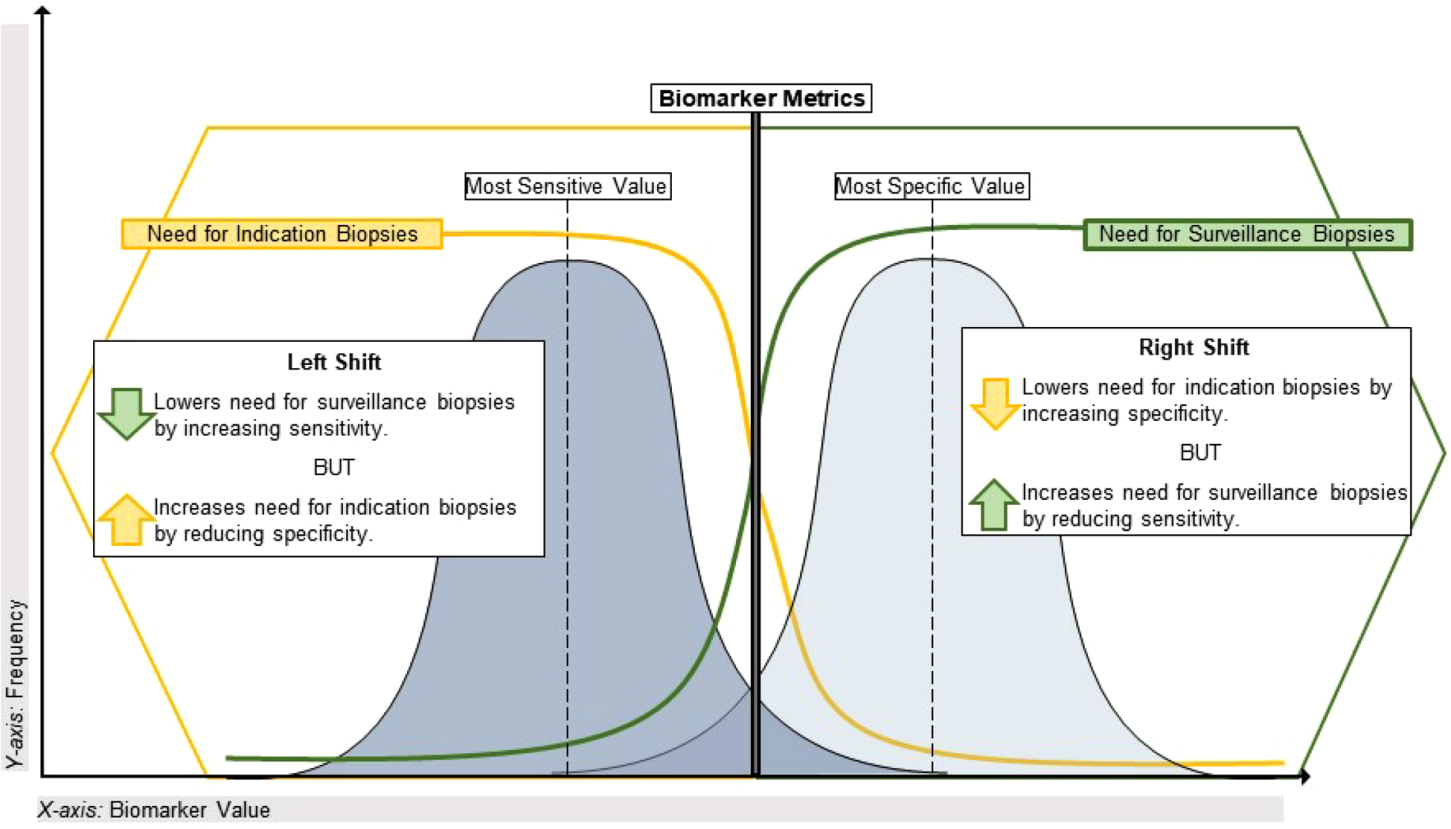
Using performance metrics to optimize application of biomarkers in transplant. This illustrates the sensitivity vs. specificity tradeoff for biomarkers of alloimmune injury as it pertains to the specific clinical dilemma of maximizing the diagnostic yield from indication and surveillance biopsies. On the left side of the plot, a highly sensitive biomarker (often with a lower value on a continuous scale) will have a lower false negative rate and a high negative predictive value given the relatively low incidence of alloimmune injury discovered in universal surveillance biopsy programs during year-1 post-transplant. A biomarker with these characteristics could reduce the need for a universal surveillance biopsy program by identifying high-risk patients for early acute rejection that would likely be missed by current non-invasive standards such as creatinine. Conversely, on the right side of the plot a highly specific biomarker (often with a higher value on a continuous scale) will have a lower false positive rate and a higher positive predictive value, given the greater incidence of clinical acute rejection discovered by for-cause/indication biopsies. With a short turnaround time, such a biomarker could reduce the need for indication biopsies in patients with allograft dysfunction if used to identify stable patients at low risk for clinical acute rejection.

Aside from the AUROC, the aforementioned biomarker performance metrics all require selection of a cutoff value that classifies patients as positive or negative. If desired, the optimal cutoff value that maximizes both sensitivity and specificity can be determined using the maximum value of the Youden index, defined as the sensitivity + specificity - 1 for each potential cutoff value for a given biomarker. However, it is more common that a cutoff value must be chosen by the investigators to prioritize either sensitivity/NPV or specificity/PPV, depending on the intended context of use. For example, some pediatric kidney transplant centers perform universal surveillance biopsies at pre-specified time points to detect subclinical acute rejection in patients with stable eGFR. Most of these patients are expected to have normal/stable biopsies, but 25%–35% will have subclinical rejection that is associated with inferior long-term outcomes (12, 13, 57). In this scenario, the goal might be to avoid surveillance biopsies in patients with a high probability of being stable, which would require a diagnostic biomarker with high sensitivity and NPV to misdiagnose and biopsy a low number of truly stable patients. Another context would be a decision to biopsy (or empirically treat) pediatric kidney transplant recipients with allograft dysfunction. In this scenario, the prevalence of clinical acute rejection is expected to be higher such that the goal might be to target biopsies to patients at high risk for rejection based on allograft dysfunction and a positive biomarker result. This would require a diagnostic biomarker with high specificity and PPV to prevent truly rejecting patients from going undiagnosed and untreated. A visual representation of this continuum is presented in Figure 1.

Ideally, a high-performing diagnostic biomarker would yield high sensitivity, specificity, NPV, and AUROC with a modest PPV given the low prevalence of acute rejection. However, this might require multiple cutoff points for a given biomarker to achieve different performance metrics depending on the context of use. Blydt-Hansen et al. used this approach in their recent report of two cutoff values for urine levels of C-X-C motif chemokine ligand 10 (CXCL10, normalized to urine creatinine) to diagnose acute rejection: a “low” threshold with a low false negative rate and a “high” threshold with a low false positive rate (47). This could be a model approach for maximizing the diagnostic utility of a given biomarker in clinical decision-making. In an ideal state, high-performing diagnostic biomarkers would also be available, affordable, reproducible, trendable, have a quick turnaround time, and be able to delineate different types of disease processes under investigation (e.g., ABMR vs. TCMR as well as rejection vs. stable) (54, 58, 59). Unfortunately, both the current gold standard biomarkers for alloimmune injury (e.g., creatinine-based eGFR and proteinuria) and many emerging novel biomarkers do not satisfy all of these ideal criteria. Ultimately, it is less likely that a single biomarker will satisfy this ideal state, requiring the coordinated use of multiple biomarkers as part of a comprehensive panel that performs closer to the ideal state than the sum of its individual components. Such approaches are being developed for blood and urine biomarkers in adult kidney transplantation (27, 60).

## 3. Subclinical acute rejection: A diagnostic entity resulting from the poor diagnostic performance of creatinine-based eGFR

Changes in serum creatinine above baseline have long been used as the gold standard biomarker of alloimmune events in kidney transplantation, and are often the basis for performing a for-cause diagnostic biopsy. Creatinine has many features of an ideal diagnostic biomarker, being affordable, reproducible, and trendable with a quick turnaround time. Unfortunately, it consistently lacks the most important feature of an ideal biomarker - strong diagnostic performance. Serum creatinine has relatively poor diagnostic sensitivity for alloimmune injury and equally poor prognostic sensitivity for graft loss (both around 50%–60%) (45). Serum creatinine levels can be altered for many non-rejection causes such as low effective circulating volume, pyelonephritis, BK viral infection, and supratherapeutic tacrolimus levels, sometimes leading to unnecessary biopsies. Conversely, it is also a relatively late biomarker for allograft injury, as a significant burden of alloimmune injury is required before clinical instability is recognized as a 20%–30% decline in eGFR. As a result, many for-cause biopsies detect more established acute rejection that is more difficult to treat. Moreover, creatinine-based eGFR is even less sensitive for detecting acute rejection in smaller pediatric recipients of adult-sized allografts, owing to the imbalance between nephron/filtration mass and creatinine-producing muscle mass (9, 61). Fluctuations in creatinine levels worsen with increasing allograft vintage, making clinical decisions based on these levels in older transplants even more challenging.

The inadequate diagnostic performance of creatinine-based eGFR has led some pediatric transplant centers to perform surveillance biopsies at pre-specified time points in presumed clinically stable patients in an effort to diagnose “subclinical rejection.” Multiple cohort studies from these universal surveillance programs have estimated the incidence of early subclinical rejection (before 6 months post-transplant) at 25%–37% (12, 44, 62, 63). Subclinical rejection is often characterized as a distinct phenotype from clinical rejection with allograft dysfunction. However, as with clinical acute rejection, subclinical rejection has been associated with increased rates of subsequent *de novo* DSA, clinical acute rejection episodes, and allograft failure (62, 15). Subclinical rejection is most often reported in the first 6 months post-transplant, but some programs perform later surveillance biopsies and detect subclinical rejection as late as 5 years post-transplant in clinically stable patients (64). Moreover, subclinical and clinical rejection phenotypes such as TCMR have highly correlated Banff injury lesion scores and similar impacts on long-term outcomes (65, 66). As such, it is more likely that subclinical and clinical rejection represent the same alloimmune process that is inadequately detected using current standard biomarkers such as creatinine-based eGFR.

Briefly, other biomarkers in clinical use including proteinuria and detection of *de novo* DSA have similar issues as creatinine when used in isolation. Recently, Naesens et al. showed in a large adult cohort >1,500 patients that proteinuria (>1 g/24 h) at 1-year post-transplant was associated with a 2-fold increased risk for 5-year allograft failure and had 85% specificity for graft failure and microcirculation injury in both surveillance and for-cause biopsies. However, the sensitivity and PPV of proteinuria for late allograft failure were much lower at 16% and 26%, respectively (49). Recent studies of molecular histology of ABMR showed that *de novo* DSA was falsely negative in up to 50% of biopsies with ABMR, highlighting its relatively poor sensitivity despite being a major Banff diagnostic criterion for ABMR (28, 42). However, a recent pediatric cohort study identified *de novo* DSA as an independent prognostic biomarker for subsequent clinical acute rejection but not allograft failure (43). Improved diagnostic biomarkers are an urgent unmet need that would allow for more reliable detection and timely interventions for acute rejection, regardless of concurrent changes in eGFR and other clinical parameters that may have no substantial impact on long-term outcomes. In the next section, we will review some of the most promising blood- and urine-derived biomarkers of acute rejection that are nearest to implementation in the clinical realm.

### 3.1. Blood-based biomarkers: Donor-derived cell-free DNA and peripheral blood gene expression profiling

#### 3.1.1. Donor-derived cell-free DNA

Donor-derived cell-free DNA (dd-cfDNA) has recently emerged as a novel diagnostic and prognostic biomarker of alloimmune events both adult and pediatric kidney transplantation. Briefly, a peripheral blood sample from the recipient is subjected to next-generation sequencing using highly polymorphic single nucleotide polymorphisms to quantify dd-cfDNA without needing to genotype the donor (17). Two commercially available dd-cfDNA assays are available at present: AlloSure® (CareDx, Brisbane, CA) and Prospera® (Natera, Austin, TX). dd-cfDNA is typically expressed as a proportion of the total circulating cfDNA in plasma, and reflects the relative amount of intracellular DNA released from an injured allograft into the recipient's circulation. dd-cfDNA is often increased at the time of diagnosis of rejection and some other forms of allograft injury, but has also been shown to elevate weeks or months prior to the detection of allograft injury by traditional methods (67). The half-life of cfDNA in the blood is less than 60 min, so changes in dd-cfDNA could be a dynamic indicator of recent allograft injury (68). Following ischemia-reperfusion injury at the time of transplantation, dd-cfDNA levels initially spike and then fall to a plateau around 4–6 weeks post-transplantation without much natural variability throughout allograft vintage thereafter (29).

Studies in adults have consistently used a cutoff dd-cfDNA of >1% to diagnose acute rejection and its TCMR/ABMR phenotypes. One of the earliest studies was the Circulating Donor-Derived Cell-Free DNA in Blood for Diagnosing Acute Rejection in Kidney Transplant Recipients (DART) study, which studied plasma dd-cfDNA fraction in 102 adult kidney transplant recipients with a 26% incidence of acute rejection (roughly split between ABMR and TCMR). The DART investigators found that dd-cfDNA fraction had superior diagnostic performance to creatinine as a biomarker of acute rejection, with an AUROC of 0.74 for dd-cfDNA vs. 0.54 for creatinine. dd-cfDNA > 1% performed especially well in diagnosing ABMR, with a sensitivity, specificity, PPV, and NPV of 81%, 83%, 44% and 96%, respectively, with an AUROC of 0.87 (25). The DART study identified dd-cfDNA as a sensitive biomarker of acute rejection but with a considerable rate of false positive tests, favoring its use as a “rule-out” biomarker to identify stable allografts without need of surveillance biopsy.

One of the larger studies appraising dd-cfDNA is the Assessing Donor-derived cell-free DNA Monitoring Insights of kidney Allografts with Longitudinal surveillance (ADMIRAL) study, in which the plasma dd-cfDNA fraction was monitored prospectively over 3 years in 1,000 adult kidney transplant recipients. Around 200 biopsies were performed, with about half being for-cause and half surveillance. The investigators used a lower cutoff for dd-cfDNA (>0.5%) and found an AUROC for all rejection of 0.80 compared to 0.49 for creatinine, but unlike the DART study found equivalent diagnostic performance for ABMR and TCMR. Moreover, the ADMIRAL study evaluated dd-cfDNA as a prognostic biomarker and found that its elevation was associated with a near two-fold increased risk for a 25% decline in eGFR or development of *de novo* DSA by 3 years post-transplant. Conversely, persistently low dd-cfDNA correlated strongly with allograft quiescence, defined as the absence of tacrolimus toxicity, BK viremia, DSA, urinary tract infection, proteinuria, allograft rejection, or recurrent glomerular disease (30).

Finally, findings from the recent Trifecta study suggest that diagnostic performance can be improved by measuring the dd-cfDNA fraction as well as the absolute quantity of circulating dd-cfDNA measured in copies/ml. In Trifecta (*n* = 367 adults), patients with dd-cfDNA > 1% or absolute dd-cfDNA > 78 copies/ml had a sensitivity of 74%, specificity of 81%, PPV of 71%, NPV of 83%, and AUROC of 0.82 for diagnosing clinical acute rejection by traditional histology. The authors speculated that the improved diagnostic performance of the two-threshold algorithm was related to dd-cfDNA quantity being more sensitive to acute rejection where systemic inflammation causes high total cfDNA levels and dd-cfDNA fraction being more sensitive in cases where acute rejection was the primary source of inflammation (28, 31).

Studies of dd-cfDNA in pediatric kidney transplantation have been sparse. Sigdel et al. studied dd-cfDNA fraction in 178 patients (including 35 children) with biopsy-matched plasma samples. While the pediatric subgroup could not be analyzed separately due to the absence of acute rejection, the overall study found dd-cfDNA to have an AUROC of 0.87 for all rejection phenotypes and no differential performance in ABMR vs. TCMR (17). Notably, they also examined subgroups with surveillance and for-cause biopsies, which had similar performance metrics as the overall cohort. Puliyanda et al. studied 67 children (33 with biopsy-matched blood samples) and found that dd-cfDNA > 0.88% was associated with *de novo* DSA with 73% sensitivity, 83% specificity, and an AUROC of 0.80. Of interest, they studied a subgroup (*n* = 5) that underwent serial monitoring of dd-cfDNA and were biopsied if the dd-cfDNA fraction eclipsed 1%. All 5 patients had no clinical suspicion of alloimmune injury, yet all were diagnosed with subclinical ABMR or mixed rejection based on dd-cfDNA results alone. In those with clinical dysfunction that were biopsied (*n* = 28), they reported a sensitivity and specificity for acute rejection of 88% and 100%, respectively. These metrics were similar to the overall cohort, with the caveat that only 4 biopsy-correlated dd-cfDNA results were in the group without rejection, so this cohort was not representative of the overall transplant population (32). Steggerda et al. recently studied serial dd-cfDNA measures in a cohort with acute rejection (*n* = 18), and detected a trend for the dd-cfDNA fraction decreasing with treatment in patients with TCMR but not with ABMR (69). Most recently, Dandamudi et al. analyzed 290 banked plasma samples from 57 pediatric kidney recipients. They found that dd-cfDNA takes about 4 months to reach a baseline level, after which a diagnostic threshold of > 1% discriminated TCMR (subclinical or clinical) from stable biopsies with an AUROC of 0.82 (compared to 0.53 for creatinine) (29).

Some drawbacks of dd-cfDNA should be considered, including its superior performance for ABMR rather than TCMR in some studies (25). This is suboptimal considering that TCMR is more common than ABMR in children. dd-cfDNA also has a long turnaround time of 48–72 h, rendering it less useful for decision support around a for-cause biopsy vs. a surveillance biopsy. Testing for dd-cfDNA is not available worldwide and remains quite costly compared to poorer performing clinical biomarkers such as serum creatinine and proteinuria. While this may limit its utility as a longitudinal biomarker until the cost is reduced, it still is less expensive than a surveillance biopsy event at many centers when one considers the cost of hospital admission, sedation, physician fees, and pathology. Lastly, dd-cfDNA is more difficult to interpret in repeat transplants and in younger/smaller patients owing to an admixture of dd-cfDNA from the prior and present kidney, and the potential for a larger proportion of baseline dd-cfDNA from a large adult kidney, respectively. While these initial pediatric data are exciting, larger representative cohort studies of the diagnostic and prognostic performance of dd-cfDNA in pediatric kidney transplantation remain an unmet need.

#### 3.1.2. Peripheral blood gene expression profiling

Peripheral blood gene expression profiling (GEP) uses patterns of mRNA abundance in the recipient's circulating leukocytes as biomarkers of alloimmune events (70). Unlike dd-cfDNA, which reflects direct injury to cells in the allograft, GEP reflects the relevant mechanisms and pathways involved in the recipient's immune response (or lack thereof) to the allograft. In brief, the recipient's whole blood is processed into total mRNA and subjected to whole genome microarray analysis to measure transcript abundance, after which bioinformatic pipelines are applied to select a group of highly informative genes that are diagnostic for a number of transplant phenotypes, including acute rejection. In the Clinical Trials in Organ Transplantation (CTOT)-08 study, Friedewald and colleagues used this approach to develop a 57-gene blood-based biomarker of subclinical acute rejection in a prospective adult cohort (*n* = 382). The GEP biomarker had specificity of 87%, NPV of 88%, and an AUROC of 0.84; this level of performance held up in two external validation cohorts and in a subsequent early access program after commercialization of the biomarker under the brand name TruGraf® (Eurofins/Transplant Genomics, Framingham, MA) (26, 33).

Subsequent studies have utilized the high specificity and NPV of this biomarker to avoid indiscriminate surveillance biopsies in presumed stable patients rather than diagnose subclinical rejection (34). Notably, the investigators included borderline TCMR in the subclinical rejection group, whereas other biomarker studies have relegated this common phenotype to the normal/stable group (45), despite consistent evidence that borderline TCMR is associated with poorer long-term outcomes (12, 13, 51, 71). To date, there have been no pediatric studies of this biomarker. A recent study showed improved diagnostic performance when GEP was paired with an in-house dd-cfDNA test (OmniGraf®, Eurofins/Transplant Genomics, Framingham, MA). Specifically, GEP and dd-cfDNA individually had NPV of 82%–84%, PPV of 47%–56%, balanced accuracy of 64%–68%, and AUROC of 0.72–0.75. When GEP and dd-cfDNA were both negative, NPV increased to 88%; more impressively, when GEP and dd-cfDNA were both positive, PPV increased to 81%. The two tests agreed (positively or negatively) in 70% of patients. GEP was significantly better at detecting TCMR whereas the dd-cfDNA was better at detecting ABMR, demonstrating the synergy of using the two biomarker platforms together (27). A summary of performance data for blood-based biomarkers is presented in Table 2.

**Table 2:** Summary of performance metrics for select biomarkers in kidney transplantation. Many of the novel biomarkers discussed in this review are presented below with a range of data on performance metrics from multiple studies in kidney transplant recipients, with corresponding references in the far right column. Similar data on clinical standards eGFR and proteinuria are presented at the bottom for comparison.

Biomarker	Sensitivity	Specificity	PPV	NPV	AUROC	Reference Number
Plasma dd-cfDNA	0.33–0.89	0.71–0.96	0.50–0.71	0.83–0.95	0.74–0.87	(25, 28, 30, 31) (17*, 29*, 32*)
Gene Expression Profiling	0.38–0.91	0.73–0.94	0.35–0.83	0.81–0.97	0.72–0.95	(26, 27, 33, 34)
Urine CXCL9	0.86	0.64–0.80	0.68–0.71	0.92	0.78–0.87	(35, 36*, 72)
Urine CXCL10	0.59–0.86	0.60–0.90	0.68–0.71	0.85–0.99	0.79–0.83	(35, 36, 73*, 37*, 38*, 47*, 72, 47)
Urinary mRNA	0.67–0.71	0.80–0.82	0.62–0.65	0.83–0.85	0.73–0.85	(19, 39)
eGFR/Creatinine	0.67	0.65	0.39	0.85	0.74	(17*)
Proteinuria	0.21–0.32	0.85–0.91	0.26–0.61	0.91	0.64–0.77	(11)

* Represent biomarker studies that included children.

### 3.2. Urine-based biomarkers: Chemokines and gene expression profiles

Urine poses a naturally advantageous source for non-invasive biomarker innovation in alloimmune kidney transplant injury. Urine is useful as it is the least invasive of the non-invasive biomarkers, especially important in pediatrics, and may be more specific for intragraft processes compared with blood-based biomarkers. Broad categories of urine-based biomarkers include protein abundance (e.g., cytokines and chemokines), mRNA expression, and metabolomic patterns concentrations, whether in isolation or in combination as a biomarker panel. As opposed to the blood-based biomarkers in the previous section, certain urine biomarkers could be assayed in real time for use in the clinic setting near the point-of-care.

#### 3.2.1. Urinary chemokines

CXCL9 and CXCL10 are a pair of urinary proteins that have been studied diligently in adult and pediatric kidney transplantation (74). These are interferon gamma-inducible urinary chemokines that are highly correlated with one another and found in multiple studies to be expressed in the setting of acute kidney transplant injury, more specifically tubulitis and interstitial inflammation in the setting of allograft rejection (35, 75). Specifically, the CTOT-01 study showed that urine CXCL9 and CXCL10 protein levels had strong diagnostic performance for Banff >= IA TCMR, with sensitivity of 74%–85%, PPV of 68%–71%, and AUROC of 0.77–0.86. The CTOT investigators also found in many cases that elevated urine CXCL9 levels were detectable up to 30 days prior to the onset of clinical dysfunction or acute rejection, and that low 6-month urine CXCL9 levels identified patients at low risk for subsequent acute rejection and loss of eGFR by 18 months post-transplant, highlighting its potential use as a susceptibility/risk biomarker. Conversely, they also found that low urinary CXCL9 during acute graft dysfunction (according to creatinine) ruled out acute rejection with NPV of 92%. Finally, urine CXCL9 and CXCL10 levels were strongly correlated with Banff tubulitis and interstitial inflammation scores and decreased with treatment of acute rejection (19).

Subsequently, the CTOT-09 study further validated the use of urine CXCL9 protein as a susceptibility/risk and diagnostic biomarker, as levels began to increase weeks prior to the development of acute rejection in presumed low-risk patients on a tacrolimus withdrawal protocol (23). Taken together, these data identified multiple contexts of use for urine CXCL9: as a risk/susceptibility biomarker, a diagnostic biomarker, a monitoring biomarker, and a prognostic biomarker. In addition, urine CCL2 [also known as monocyte chemoattractant protein-1 (MCP-1)] at 6 months post-transplant had a modest association with early (6-month) subclinical rejection and progression of chronic allograft injury lesions such as interstitial fibrosis/tubular atrophy (IFTA), with AUROC 0.63–0.70, but was strongly prognostic for subsequent allograft failure with a PPV of 96% and AUROC of 0.87 (73, 76, 77). Subsequent studies in children and adults combined urine CCL2 and CXCL10 levels to increase their diagnostic and prognostic value for acute rejection, reduced eGFR, and allograft failure compared with either biomarker in isolation (36).

Compared to blood-based biomarkers, there have been more high-quality studies of urine chemokines in pediatric kidney transplant recipients (74). Over 10 years ago, Jackson et al. validated the use of CXCL9 and CXCL10 in both adults (*n* = 110) and children (*n* = 46) for detecting allograft inflammation and found significantly elevated levels in both acute rejection and BK virus infection (unable to distinguish these two entities), with lower levels in stable quiescent allografts and in those with interstitial fibrosis/tubular atrophy (IFTA). Urine CXCL9 and CXCL10 carried an AUROC of 0.83–0.87 for either acute rejection or BKV infection, and unlike some cytokines combining them into a diagnostic panel did not improve their performance (AUROC 0.85) (37). Other studies have tried to account for the effect of BKV infection on urine chemokine levels by including BKV status in multivariable models (38). Further validation studies for urine CXCL10 were performed by Blydt-Hansen et al. They studied urine CXCL10 in both subclinical and clinical rejection, finding that clinical TCMR episodes had the highest CXCL10 levels, followed by subclinical TCMR and borderline TCMR. Urine CXCL10 was strongly correlated with increasing acute inflammation scores, including glomerulitis, tubulitis, and interstitial inflammation. The overall diagnostic performance of urine CXCL10 for subclinical and clinical acute rejection was good, with an AUROC of 0.81–0.88 (76, 78). Blydt-Hansen et al. also used a practical approach to urine CXCL10 monitoring, with a “low” threshold yielding a low false negative rate and 90% sensitivity, and a “high” threshold with a low false positive rate and 90% specificity. The low threshold achieved 90% sensitivity at the expense of lower specificity (38%), whereas the high threshold achieved 90% specificity at the expense of lower sensitivity (46%). While the overall biomarker performance was very good (AUROC 0.76), it is notable that 45% of urine samples had urine CXCL10/creatinine that fell between these 2 thresholds in a “zone of ambiguity” and were less informative (47).

Finally, recent studies by the Sarwal Laboratory have developed a multi-biomarker urine-based panel as a diagnostic for acute rejection, which includes urine CXCL10, creatinine, total protein, clusterin, and total/methylated dd-cfDNA. This panel (QSant®, NephroSant, Brisbane, CA) was subsequently validated in a cohort of 223 adult and pediatric urine samples with a 32% prevalence of acute rejection. They found a PPV of 98%, NPV of 99%, and an AUC of 0.998, with equal performance in adult and pediatric subgroups. Based on these findings, the authors speculated that biomarker-guided decision support could have reduced the number of surveillance and for-cause biopsies by nearly 70% (39, 60). These initial data are promising but may be overfitted and remain to be validated in an independent external validation cohort of children.

#### 3.2.2. Urine gene expression profiles

Analyzing urine cell pellet-derived mRNA is technically challenging and often lacks reproducibility across laboratories. Robust urine mRNA analysis requires timely processing of urine samples to preserve and stabilize the cell-derived RNA before it is rendered useless by abundant RNA-hydrolyzing enzymes (79, 80). The Suthanthiran Laboratory uses a preamplification step and a customized amplicon to improve yield of the inherently poor quality RNA in urine; these steps may not be translatable to other laboratories despite the publication of standardized protocols (81). Previous studies have measured urine mRNA that encodes for proteins involved in allograft inflammation pathways, including CD3*ε*, perforin, granzyme B, proteinase inhibitor 9, CD103, CXCL9/MIG, CXCL10/IP-10, and CXCR3 (receptor for CXCL9) (82). Higher levels of mRNA in the urine are hypothesized to represent intragraft activation of pathways involved in TCMR and other alloimmune events. The CTOT-01 study, in addition to measuring urine CXCL9/10 protein, also measured a panel of urine mRNA including CXCL9, CXCL10, CCR1, CCR5, CXCR3, CCL5/RANTES, IL-8, perforin and granzyme B. After removing highly interdependent/collinear genes, both urine CXCL9 and granzyme B mRNA were diagnostic for TCMR and ABMR, with an AUROC 0.73–0.79 (19). The CTOT-04 study also investigated urine mRNA profiles in 220 patients with 400 biopsies. They built a 3-gene parsimonious model including urine CD3*ε*, IP-10, and 18S rRNA that had the best diagnostic performance for acute rejection (AUROC 0.85). The number of ABMR cases in this study were small, but this 3 gene signature distinguished TCMR from ABMR and otherwise normal allografts (80). There have been no published urine mRNA studies in pediatric kidney transplantation, but there is an ongoing multicenter prospective cohort study (VIRTUUS) that seeks to validate urine CD3*ε*, CXCL10/IP-10, and 18s rRNA as biomarkers of acute rejection in children, with 200 patients enrolled and over 1,000 urine samples at the time of the last update (72). A summary of performance metrics for urine-based biomarkers is presented in Table 2.

## 4. Discussion: Integration of novel biomarkers in a transformative care model

Alloimmune events such as TCMR and ABMR are the main contributors to allograft failure in children. Our current methods monitoring for alloimmune events are either invasive biopsies that must be deployed somewhat indiscriminately or clinical biomarkers such as creatinine that have poor sensitivity and specificity yet continue to be used as gold standards. While novel, non-invasive blood- and urine-based biomarkers have been validated with superior performance to creatinine in multiple studies, they have yet to be integrated into mainstream clinical practice. This review was not intended to be an all-encompassing summary of novel biomarkers in the alloimmune injury field, but rather a sampling of biomarkers with multiple independent validation studies (including some in children) that seem best suited for integration from the research arena into the clinical realm.

Barriers to the general implementation of novel biomarkers in the clinic include technical challenges surrounding turnaround time, interoperator variability, reproducibility across laboratories, and reliable diagnostic cutoff values (10, 11, 54). Many biomarker studies do not report a standard set of performance metrics that allow comparison to one another as well as the clinical gold standards. Another challenge is that borderline TCMR is the most common phenotype of allograft inflammation with a consistent association with poor long-term outcomes, yet there is no consensus on how to consider it in biomarker studies. Borderline TCMR has been considered as a rejection phenotype in some (26) but a normal/stable phenotype in others (45). We propose that borderline TCMR and other “suspicious” phenotypes should be treated as a distinct category, such that diagnostic classifiers can be built using higher-grade rejection phenotypes then applied to borderline cases to determine which ones are “normal-like” that can be observed without treatment and which are “rejection-like” and require immunosuppressive therapy to avoid consequences of subsequent *de novo* DSA and rejection. Implementation in pediatric kidney transplant practice is critically important, given the greater insensitivity of creatinine and other clinical standards for allograft rejection compared to adults. However, the few pediatric biomarker studies that have been performed are often retrospective cohort studies with limited statistical power rather than the large multicenter prospective studies our field requires.

Due to a lack of randomized controlled trials, we are further away from an ideal scenario where transplant physicians can use biomarker-guided clinical decision support for pursuing a biopsy and personalizing how aggressively to treat an acute rejection episode (54). In pediatrics, the annual transplant volumes are comparatively low at most centers such that achieving adequate power to detect differences in important endpoints based on biomarkers will be difficult in absence of expensive and logistically challenging multicenter studies. Therefore, at present most biomarker data in pediatrics is extrapolated from adult or combined adult and pediatric studies, and may employ diagnostic cutoff values that are not as relevant in children as adults.

**Figure 2:**
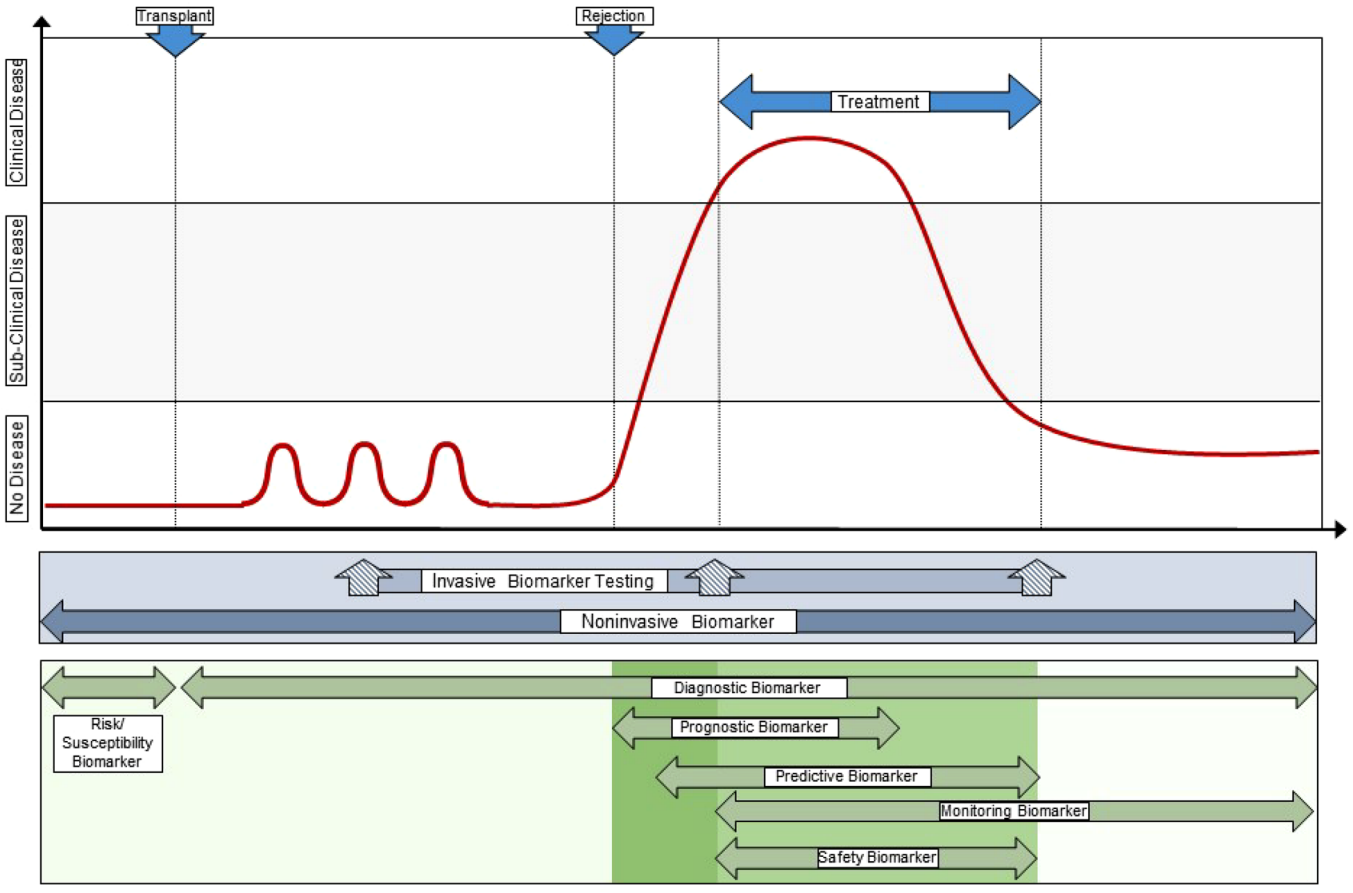
Integration of novel biomarkers with current clinical standards to transform care. This illustrates a “team approach” of using different biomarkers with different performance characteristics and context of use at different times during the post-transplant course, both in synchrony (e.g., a multi-biomarker panel) and in sequence. This approach provides personalization of alloimmune injury detection and surveillance at the individual patient level, often at the point-of-care, and allows the treating clinician to integrate far more information into decision support than any one clinical or investigational biomarker obtained in isolation. Original drawn figure Adapted with permission from Figure 3 in reference #10, Naesens et al, JASN 2018; 29(1):24–34.

Of the biomarkers reviewed here, urine CXCL9, CXCL10, and CCL2 protein have the most robust data in pediatric and young adult kidney transplantation (38, 74). There are multiple validated studies in relatively large pediatric cohorts to support their context of use as diagnostic, susceptibility/risk, monitoring, and prognostic biomarkers. These are commonly measured by commercially available ELISA kits but there are automated platforms that soon may enable clinicians to measure one or more of these biomarkers in urine with high reproducibility, accuracy, and speed to enable their implementation at the point of care (47, 83). We envision a transformative care model where pediatric kidney transplant recipients soon will have real-time urinary chemokine monitoring added to creatinine and proteinuria as part of a clinic visit (Figure 2). These additional biomarkers will help clinicians decide when a surveillance biopsy can be avoided in a likely stable patient, and when an elevated creatinine is probably due to an alloimmune event that requires a diagnostic biopsy to inform clinical care (Figure 2). We expect that additional biomarkers will soon be validated in children, such as dd-cfDNA or blood/urine GEP, that will enable the development of multi-platform biomarker panels that surpass the performance of an individual biomarker for diagnosing rejection and predicting long-term outcomes.
